# Influence of polymorphisms in the *vascular endothelial growth factor *gene on allograft rejection after kidney transplantation: a meta-analysis

**DOI:** 10.12688/f1000research.27800.1

**Published:** 2021-02-10

**Authors:** Thanee Eiamsitrakoon, Phuntila Tharabenjasin, Noel Pabalan, Hamdi Jarjanazi, Adis Tasanarong

**Affiliations:** 1 Chulabhorn International College of Medicine, Thammasat University, Rangsit, Pathumthani, 12121, Thailand; 2 Environmental Monitoring and Reporting Branch, Ontario Ministry of the Environment, Conservation and Parks, Toronto, Ontario, M5T 3L9, Canada; 3 Nephrology Unit, Faculty of Medicine, Thammasat University, Rangsit, Pathumthani, 12121, Thailand

**Keywords:** VEGF polymorphisms, allograft, renal, kidney transplantation, meta-analysis

## Abstract

**Background: **Reported associations of allograft rejection in kidney transplant patients with *VEGF* single nucleotide polymorphisms (SNPs) have been inconsistent between studies, which prompted a meta-analysis to obtain more precise estimates.

**Methods:** Using the PICO elements, kidney transplant patients (P) were compared by genotype data between rejectors (I) and non-rejectors (C) in order to determine the risk of allograft rejection (O) attributed to the *VEGF* SNPs. Literature search of four databases yielded seven articles. To calculate risks for allograft rejection, four SNPs were examined. Using the allele-genotype model we compared the variant ( *var*) with the wild-type ( *wt*) and heterozygous ( *var*- *wt*) alleles. Meta-analysis treatments included outlier and subgroup analyses, the latter was based on ethnicity (Indians/Caucasians) and rejection type (acute/chronic). Multiple comparisons were corrected with the Bonferroni test.

**Results:** Five highly significant outcomes (P ^a^ < 0.01) survived Bonferroni correction, one of which showed reduced risk for the *var* allele (OR 0.61, 95% CI 0.45-0.82). The remaining four indicated increased risk for the *wt* allele where the chronic rejection (OR 2.10, 95% CI 1.36-3.24) and Indian (OR 1.44, 95% CI 1.13-1.84) subgroups were accorded susceptibility status.

**Conclusions:** Risk associations for renal allograft rejection were increased and reduced on account of the *wt* and *var* alleles, respectively. These findings could render the *VEGF* polymorphisms useful in the clinical genetics of kidney transplantation.

## Abbreviations

 A, adenine; AR, acute rejection; C, cytosine; CA or C/A, cytosine/adenine; CEU, European population; CI, confidence interval; CR, chronic rejection; CRAD, chronic renal allograft dysfunction; C/T, cytosine/thymine; du, duplicate; G, guanine; GIH, Gujarati Indian population; GG or G/G, guanine/guanine; *het*, heterozygous genotype; HWC, Hardy-Weinberg Compliant HWE, Hardy-Weinberg Equilibrium; I ^2^, measure of variability; ITU, Telugu Indian population; KT, kidney transplantation; LD, linkage disequilibrium; n, number of studies; NRJ, non-rejection; OR, odds ratio; P ^a^, P-value for association; P _het_, P-value for heterogeneity; [R], reference of studies; RJ, rejection; SNP, single nucleotide polymorphism; T, thymine; *var*, variant allele or genotype; *VEGF*, *vascular endothelial growth factor* gene; VEGF, vascular endothelial growth factor protein; *wt*, wild-type allele or genotype

## Introduction

Chronic kidney disease is a longstanding global health problem with substantial effects on morbidity and mortality ^1^. Even with medical intervention, the likely endpoints in the progression of this disease are end-stage renal disease and kidney failure. In such cases, kidney transplantation (KT) is the current best available therapeutic option ^1, 2^. Success of the transplanted organ or an allograft in the recipient is limited by graft rejection ^3^ which is characterized by inflammatory responses toward the graft tissue resulting in structural and functional impairments leading to allograft dysfunction ^4^. Allograft rejection can be categorized largely into acute rejection (AR) which occurs days/weeks up until three months post-KT, or chronic rejection (CR) which is seen as progressive loss of graft function after three months post-KT ^5^. Key factors that contribute to allograft rejection may involve cytokines that are secreted by immune cells and antibodies against graft antigens ^6^. Cytokines have been recognized as potent immunomodulatory biomolecules that mediate physiological and pathological immune responses. These molecules determine the magnitude of alloimmune responses after transplantation, which influence graft survival ^7^. Differences in genetic background of transplant recipients are, in part, the cause of varying immune responses towards grafts ^8^. Recognizing these genetic differences and their effects on the immune response may help establish individualized immunosuppressive regimens that can improve allograft outcome ^9^. This is accomplished by identifying the alleles that may increase risk or confer protection for immune-mediated complications after KT ^10^. Single nucleotide polymorphisms (SNPs) in the cytokine genes may impact graft survival by altering transcriptional activities and levels of gene expression ^11^ which lead to variations in cytokine production ^12^.

Of the cytokine factors related to immune-mediated renal graft injury, the vascular endothelial growth factor (VEGF) is of potential use as a post-transplantation biomarker ^13^. As mediator of vascular formation, VEGF promotes endothelial cell proliferation, differentiation and survival ^14^. It also mediates endothelium-dependent vasodilation and maintains vascular permeability ^15^. Dysregulations of *VEGF* expression are evident in many renal abnormalities ^16, 17^. This suggests a possible pathologic role of this protein in renal diseases including graft injury. Studies of allograft tissues from rat KT models (in both AR and CR events) and human KT recipients with AR showed increased VEGF expression in renal tubules and interstitium ^18, 19^. This suggests involvement of this gene/protein in the pathogenesis of allograft rejection. Various SNPs in the *VEGF* gene have been identified ^20, 21^ and reported to be associated either with low or high VEGF protein production ^21, 22^. One of the common *VEGF* SNPs, a cytosine (C) to adenine (A) polymorphism at position 2578 within the promoter region (-2578 C/A), was found to be associated with VEGF expression and allograft rejection. The CC genotype was associated with high VEGF production but varied in its effects on renal allograft outcomes with reduced ^23^ and increased ^24^ rejection risks across the studies. Given the varied influence of these SNPs on renal allograft function, it is opportune to statistically synthesize these study findings using meta-analysis.

Our study aims to provide better understanding of the genetic role of *VEGF* SNPs on post-KT allograft outcome in term of risk for allograft rejection among recipients, which might guide potential future directions in transplant genetics. To obtain less ambiguous, clearer estimates of the *VEGF* role in this investigation, we apply meta-analysis techniques (i.e. outlier treatment) in order to strengthen the evidence.

## Methods

### Selection of studies

We searched for association studies on 13 February 2020, the start date for this meta-analysis. Four strings of search terms were used that included combinations of *“vascular endothelial growth factor”, “VEGF”, “polymorphism”, “cytokine”, “renal”, “transplant”, “allograft”, and “kidney transplantation”* as medical subject heading and text in MEDLINE using PubMed, Google Scholar, Science Direct and Mednar, unrestricted by language. Details of the search strategies for each of these four databases are shown in Table S1 ( *Extended data* ^25^).

References cited in the retrieved articles were also hand-screened to identify additional eligible studies. In case of duplicate articles, we selected the one with a later date of publication.

The following PICO elements were applied in the meta-analysis: (i) Population: renal allograft patients; (ii) Intervention: *VEGF* gene polymorphisms; (iii) Comparators: rejectors (RJ) versus non-rejectors (NRJ); and (iv) Outcome: allograft rejection post-KT.

Inclusion criteria were: (i) case–control design evaluating the association between *VEGF* SNPs and risk of allograft rejection; (ii) available *VEGF* genotype frequencies in the presence and absence of allograft rejection and (iii) sufficient genotype frequency data to enable calculation of the odds ratios (ORs) and 95% confidence intervals (CIs). Exclusion criteria were studies that: (i) did not involve renal allografts; (ii) were review articles; (iii) were functional studies; (iv) did not involve *VEGF* SNPs and with genotype or allele frequencies that were unusable/absent or, when available, combined with SNPs in other genes, preventing proper data extraction.

### SNP groupings

We examined four SNPs ( Table 1; *Extended data:* S2 Table ^25^). Observed phenotypic associations have been attributed to the proximity of SNPs in the *VEGF* gene ^26– 28^, termed linkage disequilibrium (LD). LD is the correlation between alleles located near each other ^29^ and is measured in terms of D′ and r ^2^ with a value of 1 indicating complete LD ^30, 31^. LD values were based on the European (CEU), and the Indian populations (Gujarati: GIH and Telugu: ITU) from LDlink. Complete LD between rs699947 (-2578C/A) and rs144854329 (-2549 insertion/deletion) merited combination, labeled VEGF1. -1154G/A (rs1570360), and 938C/T (rs3025039) were not in complete LD, thus analyzed separately, notated as VEGF2 and VEGF3, respectively ( Table 2).

**Table 1.** Characteristics of the included studies in *VEGF* meta-analysis.

First author	[R]	Year	Country	Ethnicity	Age (y) mean ± SD	Comparisons (/: versus)	*VEGF* polymorphisms (KT outcome) n	Clark- Baudouin score
Mittal	39	2011	India	Indian	36.1 ± 10.2	RJ / NRJ	rs699947, rs1570360 (AR) 2	10
Prakash	40	2015	India	Indian	37.1 ± 9.4	AR / NRJ	rs699947, rs1570360, rs3025039, rs144854329 (AR) 4	5
Prakash	41	2018	India	Indian	38.2 ± 11.6	Graft failure / functioning graft	rs699947, rs1570360, rs3025039, rs144854329 (CR) 4	6
Gunesacar	42	2007	Germany	Caucasian	31.7 ± 0.7	Graft failure / functioning graft	rs3025039 (AR) 1	6
Jimenez- Sousa	43	2012	Spain	Caucasian	50.5 (16.6) *	CRAD / non- CRAD	rs699947 (CRAD-CR) 1	6
Lemos	23	2005	Netherlands	Caucasian	47.1 ± 13.5	AR / Non-AR	rs699947, rs1570360, rs25648 (AR) 3	7
Shahbazi	24	2002	United Kingdom	Caucasian	39.0 ± 15.3	RJ / NRJ	rs699947, rs1570360 (AR) 2	6

*VEGF*: vascular endothelial growth factor; [R]: Reference; y: years; KT: kidney transplantation; RJ: rejection NRJ: non-rejection; AR: acute rejection; CR: chronic rejection; CRAD: chronic renal allograft dysfunction; n: number of studies; * median (range)

**Table 2.** Quantitative features of the included *VEGF* studies that examined associations with kidney transplantation outcome.

	First author	Ethnicity	AR/ CR	*VEGF* SNPs	Sample sizes			Statistical power	RJ			NRJ			Minor allele frequency	HWE P-value
					RJ	NRJ	Total	(α = 0.05; OR 1.5)	*wt-wt*	*wt-var*	*var-var*	*wt-wt*	*wt-var*	*var-var*		
**VEGF1** (rs699947+rs144854329)					**663**	**956**	1,619	**97.7** †								
1	Jimenez-Sousa	Caucasian	CR	rs699947	158	118	276	37.4	40	83	35	45	49	24	0.41	0.122
2	Lemos	Caucasian	AR	rs699947	93	267	360	38.1	21	46	26	60	133	74	0.53	0.987
3	Shahbazi	Caucasian	AR	rs699947	64	103	167	23.9	24	33	7	24	50	29	0.52	0.785
4	Mittal	Indian	AR	rs699947	du	du	----	----	10	23	11	30	71	55	0.58	0.412
5	Prakash5	Indian	AR	rs699947	76	196	272	31.4	23	31	22	38	119	39	0.50	**0.0027**
6	Prakash5	Indian	AR	rs144854329	du	du	----	----	19	34	23	39	101	56	0.59	0.591
7	Prakash8	Indian	CR	rs699947	98	174	272	35.1	13	52	33	48	98	28	0.44	0.288
8	Prakash8	Indian	CR	rs144854329	du	du	----	----	15	62	21	43	73	58	0.54	**0.041**
9	Prakash8	Indian	AR	rs699947	du	du	----	----	48	98	28	13	52	33	0.60	0.288
10	Prakash8	Indian	AR	rs144854329	54	218	272	----	58	73	43	21	62	15	0.47	**0.008**
**VEGF2** (rs1570360)					**105**	**254**	359	40.5 †								
1	Lemos	Caucasian	AR	rs1570360	du	du	----	----	47	38	8	118	119	30	0.34	0.999
2	Shahbazi	Caucasian	AR	rs1570360	61	98	159	23	33	25	3	34	43	21	0.43	0.291
3	Mittal	Indian	AR	rs1570360	44	156	200	21.5	13	16	15	48	51	57	0.53	**0.00002**
4	Prakash5	Indian	AR	rs1570360	du	du	----	----	27	31	18	35	115	46	0.53	**0.013**
5	Prakash8	Indian	CR	rs1570360	du	du	----	----	**23**	**53**	**22**	**39**	**93**	**42**	**0.51**	0.418
**VEGF3** (rs3025039)					**265**	**290**	555	65.2 †								
1	Gunesacar	Caucasian	AR	rs3025039	265	290	555	65.1	231	31	3	230	55	5	0.11	0.423
2	Prakash5	Indian	AR	rs3025039	du	du	----	----	20	33	23	79	80	37	0.39	**0.043**
3	Prakash8	Indian	CR	rs3025039	du	du	----	----	**39**	**42**	**17**	**60**	**71**	**43**	**0.45**	0.335

VEGF1: *vascular endothelial growth factor* polymorphisms; AR: acute rejection; CR: chronic rejection; SNPs: single nucleotide polymorphisms; RJ: rejection; NRJ: non-rejection; HWE: Hardy-Weinberg Equilibrium; *wt*: wild-type; *var*: variant; du: duplicate; the 5 and 8 after Prakash indicate the last digit of publication year for these articles; values in bold indicate total sample sizes for each *VEGF* SNP group and significant departure from the HWE; † aggregate statistical power for the *VEGF* groups.

### Data extraction and Hardy-Weinberg Equilibrium (HWE)

Two investigators (TE and NP) independently extracted data and arrived at a consensus. Authors of the component articles were contacted is cases of missing data. The following information were obtained from each publication: first author’s name, year of the study, country of origin, ethnicity, age of the subjects, comparators, *VEGF* SNPs (rs number), including transplant outcome in term of type of allograft rejection and values needed to tally the Clark-Baudouin score ( Table 1). Sample sizes as well as genotype data in RJ and NRJ were also extracted along with calculated outcomes of the minor allele frequency. HWE was assessed using the application in https://ihg.gsf.de/cgi-bin/hw/hwa1.pl, HWE was reported as P-values of the controls from the Pearson's goodness-of-fit χ ^2^-square test.

### Statistical power and quality of the studies

Using the G*Power program ^32^, we evaluated statistical power. Assuming an OR of 1.5 at a genotypic risk of α = 0.05, power was considered adequate at ≥80%. Methodological quality of the included studies was assessed with the Clark-Baudouin scale ^33^. In this scale, scores of <5, 5–6 and ≥7 represent low, moderate and high quality, respectively.

### Meta-analysis

Given the hypothesis of association between *VEGF* SNPs and risk of allograft rejection following KT, we estimated the ORs with 95% CIs for each study by comparing RJ with NRJ among transplant recipients. Table 2 shows the frequencies of the variant ( *var*) and wild-type alleles, as well as *wt*- *var* or heterozygous genotype ( *het*). Non-uniformity of the variant ( *var*) allele in VEGF1 and VEGF2 warranted the use of the allele-genotype model for VEGF1 and VEGF2. On the other hand, the *var* alleles in VEGF3 (rs3025039) were uniform (all < 0.50), so the standard genetic models were suitable: (i) homozygous: *var*– *var* and *wt*– *wt* genotypes compared with *wt*– *wt*; (ii) recessive: *var*– *var* versus *het* + *wt*– *wt*; (iii) dominant: *var*– *var* + *het* versus *wt*– *wt*; and (iv) codominant: *var* versus *wt*. Using raw data for frequencies, study specific risks (ORs) of allograft rejection were estimated and pooled ORs were calculated by comparing the effects on the same baseline. Multiple comparisons were corrected with the Bonferroni test. Subgrouping was based on ethnicity (Indians/Caucasians) and type of rejection (AR/CR). High significance (P ^a^ < 0.0001) indicated strong evidence for association.

Heterogeneity in meta-analysis ^34^ was addressed with the following: (i) its presence warranted use of the random-effects model ^35^, otherwise fixed-effects model ^36^ was used; (ii) estimated with the *χ* ^2^-based Q test ^37^; (iii) quantified with the I ^2^statistic ^38^; and (iv) sources were outlier treated. Outlier treatment divided the comparisons into pre-outlier and post-outlier.

Sensitivity analysis was used to test for robustness of the summary effects. Publication bias was considered for significant (P ^a^ < 0.05) comparisons with ≥ 10 studies ^44^. Significance was set at a two-sided P-value of < 0.05, except for heterogeneity estimation, which was set at P _het_ < 0.10) ^37^. Data for the meta-analysis were analyzed using Review Manager 5.3 (Cochrane Collaboration, Oxford, England), SIGMASTAT 2.03, and SIGMAPLOT 11.0 (Systat Software, San Jose, CA).

## Results

### Search results and study features

**Figure 1.**
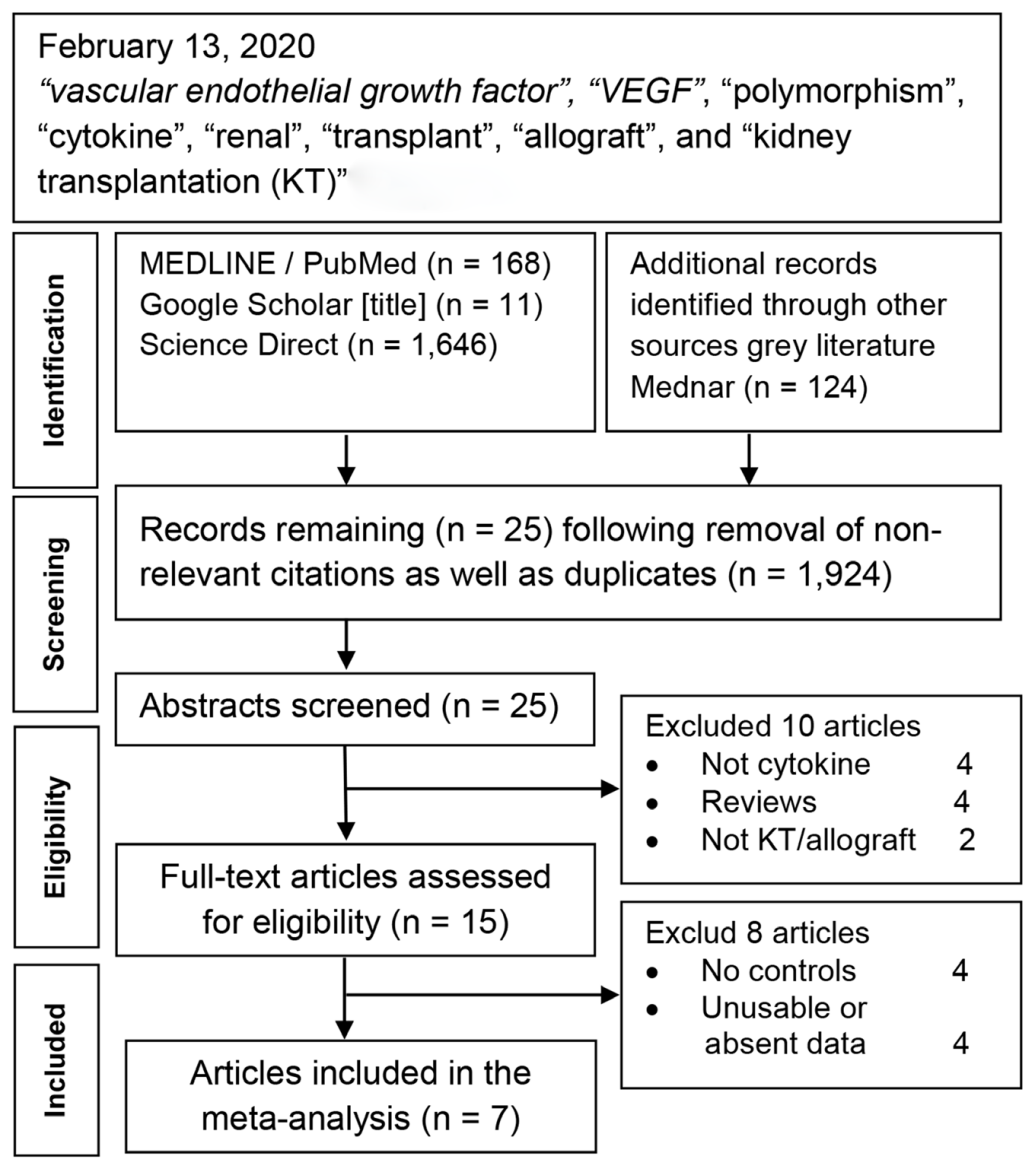
Summary flowchart of literature search.

Figure 1 outlines the study selection process in a flowchart following guidelines form the Preferred Reporting Items for Systematic Reviews and Meta-Analyses (PRISMA; *Reporting guidelines*). Table S1 ( *Extended data* ^25^) shows the initial search using combinations of four search strings applied to four databases resulted in 1,949 citations, followed by a series of omissions that mostly involved duplications (n = 1,924), The gray literature database (Mednar) yielded no additional papers for inclusion. Thus, the final number of included articles for this meta-analysis was seven ^23, 24, 39– 43^.

### Characteristics of the included studies

Of the seven articles, five ^23, 24, 39– 41^ examined more than one *VEGF* SNP ( Table 1). The number of studies VEGF1 (rs699947 and rs144854329), VEGF2 (rs1570360) and VEGF3 (rs3025039) were 10, five and three, respectively ( Table 2). Of the 10 VEGF1 studies, seven and three were in Indian ^39– 41^ and Caucasian ^23, 24, 43^ populations, respectively. Of the five VEGF2 studies, three and two were in Indian ^39– 41^ and Caucasian ^23, 24^ populations, respectively. One Caucasian ^42^ and two Indian ^40, 41^ studies comprised VEGF3. Table 1 shows two publications ^41, 43^ that investigated CR, which translated to three studies for VEGF1 ( Table 2), otherwise, the rest focused on AR ( Table 1 and Table 2).

Table 2 shows an aggregate total sample size (663 RJ/956 NRJ) and a statistical power of 97.7% for VEGF1. In contrast, both VEGF2 (105 RJ/254 NRJ) and VEGF3 (265 RJ/290 NRJ) were underpowered (40.5% and 65.2%). Mean age of the subjects was 39.96±6.6 years (± standard deviation) indicating a near to middle-age demographic profile of the KT subjects. The Clark-Baudouin scores (median 6.0, interquartile range 6.0–6.75) indicated that the methodological quality of the component studies was moderate. Control frequencies deviated from the HWE in three studies (from two articles) for VEGF1 ^40, 41^, two studies ^39, 40^ for VEGF2, and one study for VEGF3 ^40^.

### Meta-analysis outcomes

**Figure 2.**
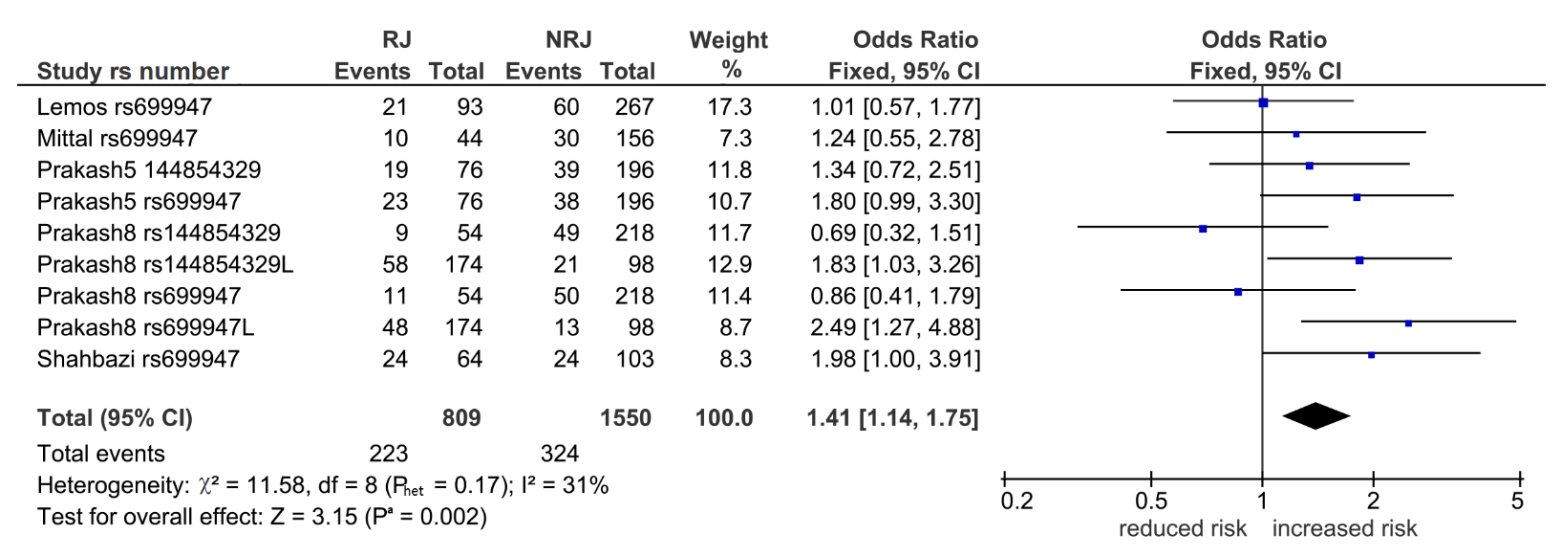
Forest plot in the post-outlier overall outcome for the *wt* allele in VEGF1 (n = 9). Diamond denotes the pooled odds ratio (OR) indicating increased risk (1.41). Squares indicate the OR in each study. Horizontal lines on either side of each square represent the 95% confidence intervals (CI). The Z test for overall effect shows significance (P ^a^ = 0.002).The χ ^2^-square test outcome has low-level heterogeneity (P _het_ = 0.17, I ^2^ = 31%). *wt*: wild-type; *VEGF*: vascular endothelial growth factor; I ^2^: a measure of variability expressed in %; RJ: rejection; NRJ: non-rejection, L: long-term

***VEGF1 associations with KT.*** Table S2 ( *Extended data* ^25^) shows 32 comparisons, six of which were significant (P ^a^ = 0.0009–0.04). Of the six, five were post-outlier derived and four survived the Bonferroni correction ( Table 3). Of the four, three were in *wt* indicating increased risk (overall: 1.41, 95% CI 1.14-1.75, P ^a ^= 0.002 [ Figure 2], Indian: OR 1.44, 95% CI 1.13-1.84, P ^a^ = 0.004, CR: OR 2.10, 95% CI, P ^a^ = 0.0009) and one in *var*, indicating reduced risk (Indian: OR 0.61, 95% CI 0.45-0.820, P ^a^ = 0.001). Only the CR outcome had zero heterogeneity (I ^2^ = 0%).

**Table 3.** Summary of main outcomes of *VEGF* SNP associations with allograft rejection post-kidney transplantation (chronic + acute).

				Test of association			Test of heterogeneity			
SNP group Genetic model	Comparison	Outlier status	n	OR	95% CI	P ^a^	P _het_	I ^2^ (%)	Analysis model	Sensitivity outcome
VEGF1										
*wt*	Overall	Post	9	1.41	1.14-1.75	0.002 *	0.17	31	Fixed	Robust
*wt*	Chronic rejection	Post	2	2.10	1.36-3.24	0.0009 *	0.50	0	Fixed	Robust
*var*	Indian	Post	5	0.61	0.45-0.82	0.001 *	0.16	39	Fixed	Robust
*wt*	Indian	Pre	7	1.44	1.13-1.84	0.004 *	0.16	35	Fixed	Robust
*var*	Overall	Post	7	0.77	0.60-0.99	0.04	0.14	37	Fixed	Not robust
*wt*	HW-compliant	Post	6	1.39	1.07-1.81	0.02	0.23	28	Fixed	Not robust
VEGF2										
*wt*	Overall	Post	4	1.58	1.19-2.09	0.001 *	0.12	49	Fixed	Robust
*wt*	Overall	Pre	5	1.48	1.01-2.15	0.04	0.09	51	Random	Not robust
*wt*	HW-compliant	Post	3	1.39	1.01-1.91	0.04	0.24	30	Fixed	Not robust
*wt*	Caucasian	Post	2	1.55	1.06-2.28	0.02	0.19	42	Fixed	Not robust
VEGF3										
*Codominant*	Overall	Post	2	0.69	0.53-0.91	0.01	0.36	0	Fixed	Not robust
*Dominant*	Overall	Post	2	0.66	0.47-0.92	0.01	0.33	0	Fixed	Not robust

*VEGF*: vascular endothelial growth factor gene; VEGF1: rs699947+rs144854329; VEGF2: rs1570360; VEGF3: rs3025039; *wt*: wild-type; *var*: variant; HW: Hardy-Weinberg; n: number of studies; OR: odds ratio; CI: confidence interval; P ^a^: P *-*value for association; P _het_: *P*-value for heterogeneity; I ^2^: measure of variability; * values in bold survived the Bonferroni correction

***VEGF2 associations with KT.*** Table S2 ( *Extended data* ^25^) shows 18 comparisons, four of which were significant (P ^a^ = 0.001–0.04), were in the *wt* model and had moderate heterogeneity (I ^2 ^= 30%-51%). Three of the four were products of pre-outlier analysis, where the HWC outcome (OR 1.39, 95% CI 1.01-1.91, P ^a^ = 0.04) confirmed the overall outcome (OR 1.48, 95% CI 1.01-2.15, P ^a^ = 0.04). The other overall outcome was post-outlier derived and survived the Bonferroni correction (OR 1.58, 95% CI 1.19-2.09, P ^a^ = 0.0001). The significant Caucasian outcome (OR 1.55, 95% CI 1.06-2.28, P ^a^ = 0.02) contrasted with the non-significant Indian outcome (OR 1.36, 95% CI 0.72-2.58, P ^a^ = 0.34).

***VEGF3 associations with KT.*** Table S3 ( *Extended data* ^25^) shows eight comparisons, two of which were significant (P ^a^ = 0.008–0.01) but did not withstand Bonferroni correction. These two homogeneous (I ^2^ = 0%) pooled ORs indicated reduced risk in the dominant and codominant models (ORs 0.66–0.69, 95% CIs 0.47-0.92).

***Summary of significant VEGF associations with KT.*** Table 3 summarizes the information on the 12 significant outcomes, five of which survived the Bonferroni correction, four in VEGF1 and one in VEGF2, all deemed robust. These outcomes identified three *VEGF* polymorphisms (rs699947, rs144854329 and rs1570360) that were associated with allograft rejection post-KT. VEGF1 subgroup outcomes identified CR associations and Indians to be at risk. Depending on the genetic model, the Indian population were both susceptible ( *wt*: OR 1.44, 95% CI 1.13-1.84) and protected ( *var*: OR 0.61, 95% CI 0.45-0.82).

## Discussion

### Summary of findings

The five Bonferroni-filtered findings ( *wt* and *var* alleles) were either products of outlier treatment and/or subgrouping. Subgrouping identified the ethnicity and rejection type that was significant, thus specifying associations of the *VEGF* polymorphisms with allograft rejection post-KT. Subgrouping provided contrasts regarding significant outcomes: (i) In VEGF1, significant in Indians (P ^a^ = 0.001–0.004), non-significant in Caucasians (P ^a^ = 0.78–1.00); (ii) in VEGF2, significant in Caucasians (P ^a^ = 0.02) and non-significant in Indians (P ^a^ = 0.34); (iii) in VEFG1, significant in CR (P ^a^ = 0.0009), non-significant in AR (P ^a^ = 0.12). Subjecting these P ^a^-values to Bonferroni correction and sensitivity treatment raised the level of evidence that facilitated interpretation with greater confidence. We have shown that meta-analytical tools such as subgrouping, outlier and sensitivity treatments are instrumental in generating evidence for association. By design, such features are not present in the component single-study outcomes. This underpins the value of meta-analysis in systematically synthesizing primary study results and providing insight into associations of *VEGF* SNPs with allograft rejection post-KT. Conflicting outcomes between primary studies may be due to small sample sizes, hence, lack of power. Underpowered outcomes appear to be common in candidate gene studies ^45^ and are prone to the risk of Type 1 error. In spite of the evidence for associations, the complexity of allograft rejection involves interactions between genetic and non-genetic factors allowing for the likelihood of environmental involvement. Gene-gene and gene-environment interactions have been reported to have roles in associations of other SNPs with post-KT allograft rejection. Two articles ^39, 43^ examined polymorphisms in other genes that included *interleukin 18* ( *IL18*), *transforming growth factor beta 1* ( *TGFB1*) and *angiotensin II receptor type 1* ( *AGTR1*) *.* None of the seven articles acknowledged gene-environment interaction. Four ^23, 39– 41^ of the included articles mentioned haplotype analysis with three presenting haplotype data ^23, 39, 40^. Additional well-designed studies exploring other parameters would confirm or modify our results in this study and add to the extant knowledge about the association of the *VEGF* SNPs and renal allograft outcome.

### VEGF and renal allografts

VEGF plays a crucial role in kidney physiology with its involvement in maintaining the integrity and permeability of the glomerular capillary basement membrane ^17^. Adaptive response of VEGF toward renal allograft tissue may be related to its angiogenic property on endothelial cells since VEGF contributes to tissue repair response of damaged capillaries ^23^. After KT, the recipient’s neutrophils and macrophages infiltrate the allograft after reperfusion of the transplanted tissue leading to the production of VEGF ^24^. Shahbazi *et al.* showed that genetically directed variations in VEGF production with increased frequency of VEGF producing alleles seemed to influence susceptibility to acute allograft rejection ^24^. However, Lemos *et al.* also suggested that renal allograft recipients with genetic potential for high VEGF production had significantly better graft survival compared to recipients with low VEGF production ^23^. Our results along the timeline of post-KT outcomes indicated increased risks, both for AR and CR in the *wt* allele, which agreed with Shahbazi *et al.* ^24^ but contrasted with Lemos *et al.* ^23^. However, the significance of our increased risk CR finding may require caution in its interpretation given the low number of studies (n = 2) and low statistical power (64.4%). More studies may be needed to clarify our CR outcome. In terms of ethnicity, Indians carriers of the *wt* CC genotype in rs699947 (-2578C/A), were afforded better graft survival than the CA and AA genotypes ^41^. In contrast, Shahbazi *et al.* found that the -2578 C allele (rs699947) and the -1154 G allele (rs1570360) were associated with increased risk of acute renal allograft rejection in Caucasians conferring greater risk among *wt* homozygotes (-2578C/C and -1154 G/G) compared to -2578C/A and -1154G/A heterozygous genotypes ^24^. These inconsistent associations among previous studies may be due to the variations in genetic background influenced by differential ethnicities of the patients.

### Strengths and limitations

Interpreting our findings should consider its limitations and strengths. Strengths include: (i) VEGF1 combined sample sizes translated to high aggregate statistical power (97.7%); (ii) significant HWC outcomes validated the overall pooled effects in *wt*. These validations served to reduce the risk of genotyping errors and minimize methodological weaknesses in our study; (iii) subgroup outcomes in CR and Indians point to potential clinical utility in the genetics of renal transplantation; (iv) efficiency of outlier treatment was the key to generating associative significance and eliminating or reducing heterogeneity and (v) stability of the core overall outcomes are underpinned by surviving the Bonferroni correction (minimizing Type 1 error risk) and robustness (determined with sensitivity treatment). On the other hand, limitations include: (i) all the component studies were underpowered; (ii) most of the moderately significant outcomes (67%) were non-robust.

## Conclusions

To our knowledge, this is the first meta-analysis to examine associations between *VEGF* SNPs and risk of allograft rejection post-KT. Risks for renal allograft rejection associated with *VEGF* polymorphisms were shown to be increased up to 1.6-fold for the *wt* allele and 39% reduced for the *var* allele. Subgroups found to be susceptible were the Indian population and CR. These highly significant and robust core effects could render the *VEGF* polymorphisms useful as a prognostic biomarker in allograft rejection post-KT.

## Data availability

### Underlying data

All data underlying the results are available as part of the article and no additional source data are required.

### Extended data

Dryad: Influence of polymorphisms in the vascular endothelial growth factor gene on allograft rejection after kidney transplantation: a meta-analysis, https://doi.org/10.5061/dryad.gqnk98skz ^25^.

This project contains the following extended data:

- **S1 Table** Overall, modified and subgroup outcomes for VEGF1.
- **S2 Table** Overall, modified and subgroup outcomes for VEGF2.
- **S3 Table** Overall, modified and subgroup outcomes for VEGF3.

### Reporting guidelines

Dryad: PRISMA checklist for ‘Influence of polymorphisms in the vascular endothelial growth factor gene on allograft rejection after kidney transplantation: a meta-analysis’, https://doi.org/10.5061/dryad.gqnk98skz ^46^.

Data are available under the terms of the Creative Commons Zero "No rights reserved" data waiver (CC0 1.0 Public domain dedication).

## References

[1] Levey AS Atkins R Coresh J : Chronic kidney disease as a global public health problem: approaches and initiatives - a position statement from Kidney Disease Improving Global Outcomes. *Kidney Int.* 2007;72(3):247–259. 10.1038/sj.ki.5002343 17568785

[2] Howard K Salkeld G White S : The cost-effectiveness of increasing kidney transplantation and home-based dialysis. *Nephrology (Carlton).* 2009;14(1):123–132. 10.1111/j.1440-1797.2008.01073.x 19207859

[3] Critchley WR Fildes JE : Graft rejection - endogenous or allogeneic? *Immunology.* 2012;136(2):123–132. 10.1111/j.1365-2567.2012.03560.x 22260525 PMC3403257

[4] Goldberg RJ Weng FL Kandula P : Acute and Chronic Allograft Dysfunction in Kidney Transplant Recipients. *Med Clin North Am.* 2016;100(3):487–503. 10.1016/j.mcna.2016.01.002 27095641

[5] Bhowmik DM Dinda AK Mahanta P : The evolution of the Banff classification schema for diagnosing renal allograft rejection and its implications for clinicians. *Indian J Nephrol.* 2010;20(1):2–8. 10.4103/0971-4065.62086 20535263 PMC2878403

[6] Seeger H Lindenmeyer MT Cohen CD : Lymphotoxin expression in human and murine renal allografts. *PLoS One.* 2018;13(1):e0189396. 10.1371/journal.pone.0189396 29300739 PMC5754061

[7] Bestard O Cruzado JM la Franquesa M : Biomarkers in renal transplantation. *Curr Opin Organ Transplant.* 2010;15(4):467–473. 10.1097/MOT.0b013e32833b9ccb 20613522

[8] Dmitrienko S Hoar DI Balshaw R : Immune response gene polymorphisms in renal transplant recipients. *Transplantation.* 2005;80(12):1773–1782. 10.1097/01.tp.0000184624.54005.9f 16378074

[9] Pascual M Theruvath T Kawai T : Strategies to improve long-term outcomes after renal transplantation. *N Engl J Med.* 2002;346(8):580–590. 10.1056/NEJMra011295 11856798

[10] Krüger B Schröppel B Murphy BT : Genetic polymorphisms and the fate of the transplanted organ. *Transplant Rev (Orlando).* 2008;22(2):131–140. 10.1016/j.trre.2007.12.002 18631866

[11] Perco P Oberbauer R : Integrative analysis of -omics data and histologic scoring in renal disease and transplantation: renal histogenomics. *Semin Nephrol.* 2010;30(5):520–530. 10.1016/j.semnephrol.2010.07.009 21044763 PMC3359618

[12] Wilson AG Symons JA McDowell TL : Effects of a polymorphism in the human tumor necrosis factor alpha promoter on transcriptional activation. *Proc Natl Acad Sci U S A.* 1997;94(7):3195–3199. 10.1073/pnas.94.7.3195 9096369 PMC20345

[13] Herath S Erlich J Au AYM : Advances in Detection of Kidney Transplant Injury. *Mol Diagn Ther.* 2019;23(3):333–351. 10.1007/s40291-019-00396-z 30941671

[14] Ferrara N : Molecular and biological properties of vascular endothelial growth factor. *J Mol Med (Berl).* 1999;77(7):527–543. 10.1007/s001099900019 10494799

[15] Ferrara N Gerber HP : The role of vascular endothelial growth factor in angiogenesis. *Acta Haematol.* 2001;106(4):148–156. 10.1159/000046610 11815711

[16] Doi K Noiri E Fujita T : Role of vascular endothelial growth factor in kidney disease. *Curr Vasc Pharmacol.* 2010;8(1):122–128. 10.2174/157016110790226606 19485913

[17] Schrijvers BF Flyvbjerg A De Vriese AS : The role of vascular endothelial growth factor (VEGF) in renal pathophysiology. *Kidney Int.* 2004;65(6):2003–2017. 10.1111/j.1523-1755.2004.00621.x 15149314

[18] Rintala SE Savikko J Rintala JM : Vascular endothelial growth factor (VEGF) ligand and receptor induction in rat renal allograft rejection. *Transplant Proc.* 2006;38(10):3236–3238. 10.1016/j.transproceed.2006.10.049 17175233

[19] Ozdemir BH Ozdemir FN Haberal N : Vascular endothelial growth factor expression and cyclosporine toxicity in renal allograft rejection. *Am J Transplant.* official journal of the American Society of Transplantation and the American Society of Transplant Surgeons.2005;5(4 Pt 1):766–774. 10.1111/j.1600-6143.2005.00772.x 15760400

[20] Brogan IJ Khan N Isaac K : Novel polymorphisms in the promoter and 5' UTR regions of the human vascular endothelial growth factor gene. *Hum Immunol.* 1999;60(12):1245–1249. 10.1016/s0198-8859(99)00132-9 10626738

[21] Watson CJ Webb NJ Bottomley MJ : Identification of polymorphisms within the vascular endothelial growth factor (VEGF) gene: correlation with variation in VEGF protein production. *Cytokine.* 2000;12(8):1232–1235. 10.1006/cyto.2000.0692 10930302

[22] Renner W Kotschan S Hoffmann C : A common 936 C/T mutation in the gene for vascular endothelial growth factor is associated with vascular endothelial growth factor plasma levels. *J Vasc Res.* 2000;37(6):443–448. 10.1159/000054076 11146397

[23] Lemos FBC Mol WM Roodnat JI : The beneficial effects of recipient-derived vascular endothelial growth factor on graft survival after kidney transplantation. *Transplantation.* 2005;79(9):1221–1225. 10.1097/01.tp.0000161219.75906.ec 15880074

[24] Shahbazi M Fryer AA Pravica V : Vascular endothelial growth factor gene polymorphisms are associated with acute renal allograft rejection. *J Am Soc Nephrol.* 2002;13(1):260–264. 11752046 10.1681/ASN.V131260

[25] Pabalan N : Influence of polymorphisms in the vascular endothelial growth factor gene on allograft rejection after kidney transplantation: a meta-analysis, *Dryad*, Dataset,2020. 10.5061/dryad.gqnk98skz PMC8905004 35284063

[26] Kapahi R Guleria K Sambyal V : Association of *VEGF* and *VEGFR1* polymorphisms with breast cancer risk in North Indians. *Tumour Biol.* 2015;36(6):4223–4234. 10.1007/s13277-015-3059-1 25604142

[27] Metzger CS Koutsimpelas D Brieger J : Transcriptional regulation of the VEGF gene in dependence of individual genomic variations. *Cytokine.* 2015;76(2):519–526. 10.1016/j.cyto.2015.07.015 26209503

[28] Scartozzi M Bianconi M Faloppi L : VEGF and VEGFR polymorphisms affect clinical outcome in advanced renal cell carcinoma patients receiving first-line sunitinib. *Br J Cancer.* 2013;108(5):1126–1132. 10.1038/bjc.2012.501 23511629 PMC3619056

[29] Borecki I : Linkage and Association Studies. Encyclopedia of Life Sciences.John Wiley & Sons, Ltd.,2001. Reference Source

[30] Lewontin RC : On measures of gametic disequilibrium. *Genetics.* 1988;120(3):849–852. 3224810 10.1093/genetics/120.3.849 PMC1203562

[31] Hill WG Robertson A : Linkage disequilibrium in finite populations. *Theor Appl Genet.* 1968;38(6):226–231. 10.1007/BF01245622 24442307

[32] Faul F Erdfelder E Lang AG : G*Power 3:a flexible statistical power analysis program for the social, behavioral, and biomedical sciences. *Behav Res Methods.* 2007;39(2):175–191. 10.3758/bf03193146 17695343

[33] Clark MF Baudouin SV : A systematic review of the quality of genetic association studies in human sepsis. *Intensive Care Med.* 2006;32(11):1706–1712. 10.1007/s00134-006-0327-y 16957907

[34] Higgins JP : Commentary: Heterogeneity in meta-analysis should be expected and appropriately quantified. *Int J Epidemiol.* 2008;37(5):1158–1160. 10.1093/ije/dyn204 18832388

[35] DerSimonian R Laird N : Meta-analysis in clinical trials. *Control Clin Trials.* 1986;7(3):177–188. 10.1016/0197-2456(86)90046-2 3802833

[36] Mantel N Haenszel W : Statistical aspects of the analysis of data from retrospective studies of disease. *J Natl Cancer Inst.* 1959;22(4):719–748. 13655060

[37] Higgins JP Thompson SG Deeks JJ : Measuring inconsistency in meta-analyses. *BMJ.* 2003;327(7414):557–560. 10.1136/bmj.327.7414.557 12958120 PMC192859

[38] Higgins JP Thompson SG : Quantifying heterogeneity in a meta-analysis. *Stat Med.* 2002;21(11):1539–1558. 10.1002/sim.1186 12111919

[39] Mittal RD Srivastava P Singh V : Association of common variants of vascular endothelial growth factor and interleukin-18 genes with allograft survival in renal transplant recipients of North India. *DNA Cell Biol.* 2011;30(5):309–315. 10.1089/dna.2010.1138 21323573

[40] Prakash S Agrawal S Kumar S : Impact of vascular endothelial growth factor single nucleotide polymorphism association on acute renal allograft rejection. *Nephron.* 2015;129(2):91–96. 10.1159/000368700 25659610

[41] Prakash S Patel MR Agrawal S : Vascular Endothelial Growth Factor Gene Polymorphism Is Associated With Long-term Kidney Allograft Outcomes. *Kidney Int Rep.* 2018;3(2):321–327. 10.1016/j.ekir.2017.10.008 29725635 PMC5932120

[42] Gunesacar R Opelz G Erken E : VEGF 936 C/T gene polymorphism in renal transplant recipients: association of the T allele with good graft outcome. *Hum Immunol.* 2007;68(7):599–602. 10.1016/j.humimm.2007.03.015 17584582

[43] Jimenez-Sousa MA Fernandez-Rodriguez A Heredia M : Genetic polymorphisms located in TGFB1, AGTR1, and VEGFA genes are associated to chronic renal allograft dysfunction. *Cytokine.* 2012;58(3):321–326. 10.1016/j.cyto.2012.02.017 22433249

[44] Ioannidis JP Trikalinos TA : The appropriateness of asymmetry tests for publication bias in meta-analyses: a large survey. *CMAJ.* 2007;176(8):1091–1096. 10.1503/cmaj.060410 17420491 PMC1839799

[45] Dumas-Mallet E Button KS Boraud T : Low statistical power in biomedical science: a review of three human research domains. *R Soc Open Sci.* 2017;4(2):160254. 10.1098/rsos.160254 28386409 PMC5367316

[46] Pabalan N : Influence of polymorphisms in the vascular endothelial growth factor gene on allograft rejection after kidney transplantation: a meta-analysis. *Dryad.* Dataset,2021. 10.5061/dryad.gqnk98skz PMC8905004 35284063

