# Supplementary material for: Influence of polymorphisms in the vascular endothelial growth factor gene on allograft rejection after kidney transplantation: a meta-analysis
Source: F1000Res. 2021 Feb 10;10:90. [Version 1] doi: 10.12688/f1000research.27800.1 (PMC8905004; doi:10.12688/f1000research.27800.1)
Supplement: Peer review history

# Peer review: Reviewer response for version 1

**DOI:** 10.5256/f1000research.30745.r119145

**Published:** 2022-03-08

**Authors:** Iman Karimzadeh

**Affiliations:** 1 Department of Clinical Pharmacy, School of Pharmacy, Shiraz University of Medical Sciences, Shiraz, Iran

The authors in this meta-analysis focused on the possible association between vascular endothelial growth factor gene polymorphism and allograft rejection after kidney transplantation. They demonstrated that risks for renal allograft rejection associated with *VEGF* polymorphisms were shown to be increased up to 1.6-fold for the wild type allele and 39% reduced for the variant allele. The topic is novel and interesting. The meta analysis was done properly.  The manuscript is well-written. Therefore, the manuscript can be considered for indexing after implementing all comments and revisions.

- The first reference related to the frequency of acute rejection in kidney transplantation should be added into the first paragraph of the introduction section.
- The second reference related to the role of urine VEGF level in allograft rejection should be added and briefly discussed in the second paragraph of the introduction section.  

Is the work clearly and accurately presented and does it cite the current literature?

Yes

If applicable, is the statistical analysis and its interpretation appropriate?

Yes

Are all the source data underlying the results available to ensure full reproducibility?

Yes

Is the study design appropriate and is the work technically sound?

Yes

Are the conclusions drawn adequately supported by the results?

Yes

Are sufficient details of methods and analysis provided to allow replication by others?

Yes

Reviewer Expertise:

Pharmacogenetics in the setting of kidney transplantation.

I confirm that I have read this submission and believe that I have an appropriate level of expertise to confirm that it is of an acceptable scientific standard.

## References

[1] : Association between the Three Polymorphisms of the Glucocorticoid Receptor Gene and the Early Clinical Outcome in Kidney Transplantation Patients. *Iran J Med Sci* .46(6) : 10.30476/ijms.2020.85872.1550 444-453 10.30476/ijms.2020.85872.1550 34840385PMC8611220

[2] : Acute renal allograft rejection is associated with increased levels of vascular endothelial growth factor in the urine. *Nephrology (Carlton)* .2008;13(1) : 10.1111/j.1440-1797.2007.00856.x 73-9 10.1111/j.1440-1797.2007.00856.x 18199108

# Peer review: Reviewer response for version 1

**DOI:** 10.5256/f1000research.30745.r121055

**Published:** 2022-02-14

**Authors:** Ileana Constantinescu, Ion Maruntelu

**Affiliations:** 1 Centre of Immunogenetics and Virology, Fundeni Clinical Institute, Bucharest, Romania; 2 Carol Davila University of Medicine and Pharmacy, Bucharest, Romania

The article written by Puntilla and colleagues is very interesting and challenging. The subject is very actual, searching for new biomarkers in attempt to maximize long survival after kidney transplantation. The approach of many research teams is to reveal new biomarkers with impact on kidney allograft rejection. The VEGF gene polymorphisms completes other discussed biomarkers with impact on acute and chronic kidney rejection, like microRNAs, anti-angiotensin II type 2 receptor antibody and urinary cytokines, MIG, IP-10 etc.

 Transplant genetics is complex. We have to have in mind genetic background of chronic renal disease and also the genetic status of transplantation per se.

 The meta analysis of VEGF SNPs is comprehensive well-documented using updated and accurate statistical methods. The literature search is well represented and well documented. The conclusions are clear. The information is valuable but reading the article could be difficult for a person who is not used with such extensive statistical analyses. I strongly recommend the indexing of this article with only one comment: it is very captivating but difficult to follow in its current format and must be converted to an easier content.

Is the work clearly and accurately presented and does it cite the current literature?

Yes

If applicable, is the statistical analysis and its interpretation appropriate?

Yes

Are all the source data underlying the results available to ensure full reproducibility?

Yes

Is the study design appropriate and is the work technically sound?

Yes

Are the conclusions drawn adequately supported by the results?

Yes

Are sufficient details of methods and analysis provided to allow replication by others?

Yes

Reviewer Expertise:

Immunology, Immunogenetics, Transplantation, Molecular Biology, Virology

We confirm that we have read this submission and believe that we have an appropriate level of expertise to confirm that it is of an acceptable scientific standard.
